# Acceptability, Usefulness, and Ease of Use of an Enhanced Video Directly Observed Treatment System for Supporting Patients With Tuberculosis in Kampala, Uganda: Explanatory Qualitative Study

**DOI:** 10.2196/46203

**Published:** 2023-11-10

**Authors:** Juliet Nabbuye Sekandi, Adenike McDonald, Damalie Nakkonde, Sarah Zalwango, Vicent Kasiita, Patrick Kaggwa, Robert Kakaire, Lynn Atuyambe, Esther Buregyeya

**Affiliations:** 1 Department of Epidemiology and Biostatistics University of Georgia Athens, GA United States; 2 Global Health Institute University of Georgia Athens, GA United States; 3 School of Public Health Makerere University Kampala Uganda; 4 Directorate of Public Health Services and Environment Kampala Capital City Authority Kampala Uganda; 5 Infectious Disease Institute Makerere University Kampala Uganda

**Keywords:** digital adherence technologies, tuberculosis, video directly observed therapy, acceptability, directly observed therapy, DOT, Uganda, treatment, digital, technology acceptance model, social support, privacy, stigma

## Abstract

**Background:**

In tuberculosis (TB) control, nonadherence to treatment persists as a barrier. The traditional method of ensuring adherence, that is, directly observed therapy, faces significant challenges that hinder its widespread adoption. Digital adherence technologies such as video directly observed therapy (VDOT) are emerging as promising solutions. However, as these novel technologies gain momentum, a critical gap is the lack of comprehensive studies evaluating their efficacy and the unique experiences of patients in Africa.

**Objective:**

The aim of this study was to assess patients’ experiences that affected acceptability, usefulness, and ease of use with an enhanced VDOT system during monitoring of TB treatment.

**Methods:**

We conducted individual open-ended interviews in a cross-sectional exit qualitative study in Kampala, Uganda. Thirty participants aged 18-65 years who had completed the VDOT randomized trial were purposively selected to represent variability in sex, adherence level, and HIV status. We used a hybrid process of deductive and inductive coding to identify content related to the experience of study participation with VDOT. Codes were organized into themes and subthemes, which were used to develop overarching categories guided by constructs adapted from the modified Technology Acceptance Model for Resource-Limited Settings. We explored participants’ experiences regarding the ease of use and usefulness of VDOT, thereby identifying the facilitators and barriers to its acceptability. Perceived usefulness refers to the benefits users expect from the technology, while perceived ease of use refers to how easily users navigate its various features. We adapted by shifting from assessing perceived to experienced constructs.

**Results:**

The participants’ mean age was 35.3 (SD 12) years. Of the 30 participants, 15 (50%) were females, 13 (43%) had low education levels, and 22 (73%) owned cellphones, of which 10 (45%) had smartphones. Nine (28%) were TB/HIV-coinfected, receiving antiretroviral therapy. Emergent subthemes for facilitators of experienced usefulness and ease of VDOT use were SMS text message reminders, technology training support to patients by health care providers, timely patient-provider communication, family social support, and financial incentives. TB/HIV-coinfected patients reported the added benefit of adherence support for their antiretroviral medication. The external barriers to VDOT’s usefulness and ease of use were unstable electricity, technological malfunctions in the app, and lack of cellular network coverage in rural areas. Concerns about stigma, disease disclosure, and fear of breach in privacy and confidentiality affected the ease of VDOT use.

**Conclusions:**

Overall, participants had positive experiences with the enhanced VDOT. They found the enhanced VDOT system user-friendly, beneficial, and acceptable, particularly due to the supportive features such as SMS text message reminders, incentives, technology training by health care providers, and family support. However, it is crucial to address the barriers related to technological infrastructure as well as the privacy, confidentiality, and stigma concerns related to VDOT.

## Introduction

Nonadherence to treatment poses a critical challenge to ending the tuberculosis (TB) epidemic and meeting the World Health Organization’s (WHO) End TB Strategy by 2030 [1,2]. Globally, there were approximately 10.6 million new cases of TB in 2021, the majority of whom resided in low- and middle-income countries [3]. Uganda is one of the highest TB/HIV-burdened countries, as designated by the WHO [4]. In 2021, the estimated incidence was 199 TB cases per 100,00 persons with a death rate of 28 per 100,000 persons [3]. Kampala, the capital city of Uganda, has one of the highest incidences of TB in the country [5]. In 1995, Uganda adopted the WHO-recommended short course of directly observed therapy, but its implementation had several challenges. For example, the requirement of daily observation while patients swallow their medications has limited feasibility because it is labor-intensive and costly to travel to health facilities or to patients’ homes. This is coupled with a severe shortage of health workers and other resources [6,7].

In 2017, the WHO endorsed the use of video directly observed therapy (VDOT) for treatment monitoring as a conditional recommendation based on limited empirical evidence [1]. VDOT is a relatively new technology-based system that allows either synchronous or asynchronous monitoring of patients while they take their medications [8]. Digital adherence technologies include 99DOTS, evriMED, and VDOT systems that utilize either feature phone, digital pillboxes, or smartphones apps, respectively, to offer patient-centered alternatives for TB treatment support and monitoring [9]. Recent studies in high-income [10-13] and low- to middle-income settings [14,15] have reported increased adherence, high levels of acceptability and feasibility, cost-savings, and cost-effectiveness when using VDOT. Studies that have specifically compared VDOT to usual care have also shown a high level of patient satisfaction with VDOT due to the flexibility and perceived privacy that it provides [12,16]. In Uganda, a pilot study with VDOT reported on the feasibility, acceptability, and satisfaction, but there was limited assessment of users’ experiences [14]. Overall, there are very few studies that have evaluated the use of VDOT in Uganda and the rest of Africa, suggesting that the published evidence of patient experiences is still limited. This paper reports patients’ experiences after using VDOT at the exit of our randomized controlled trial [17].

## Methods

### Ethics Approval

This study was approved by the institutional review boards at the University of Georgia (ID PROJECT00000571), Makerere University (protocol 756), and the Uganda National Council for Science and Technology. All participants provided written informed consent to participate and audio-record discussions during the exit interviews. Written informed consent was obtained in either English or Luganda language according to patients’ preferences. Participants were reimbursed for transportation and their time spent on the day of the interview.

### Theoretical Framework

**Figure 1:**
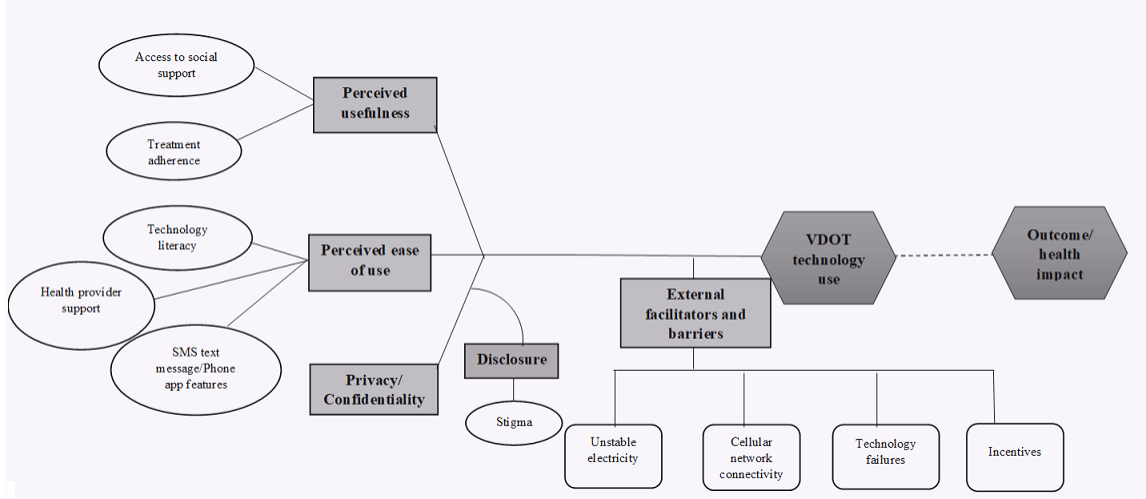
Adapted Technology Acceptance Model in Resource-Limited Settings: theoretical framework for mobile health interventions targeted to low-literacy end users in resource-limited settings [18]. VDOT: video directly observed therapy.

Our assessments and analyses were guided by constructs from the Technology Acceptance Model in Resource-Limited Settings (TAM-RLS) [18], which extended the original TAM. The original TAM model posits that motivation to accept and use technology is based on 2 main factors: perceived usefulness and perceived ease of use [19]. Perceived usefulness refers to the benefits that users think they will obtain from using the new technology. Perceived ease of use refers to the simplicity in maneuvering through the different elements of the technology [20]. Perceived usefulness and ease of use directly affect behavioral intention; they can be used to understand why users accept or refuse to use technology [21,22]. In our study, we adapted some constructs from the TAM-RLS to assess the experienced usefulness and ease of use to replace the perceived aspects. We also assessed perceived negative social influences related to privacy, confidentiality, and stigma, as shown in Figure 1. The TAM-RLS model is justified because it was developed based on an HIV-infected population with low socioeconomic status, education, and low technology experience in rural Uganda. The model also accounts for additional contextual factors that may affect the acceptability and use of technology [18]. In our study, we evaluated external facilitators and barriers that impacted the use of the VDOT technology. To our knowledge, the TAM-RLS framework has not previously been adapted to assess the experiences of patients with TB with digital health.

### Study Setting and Population

This study was conducted in Kampala, Uganda, in a population of participants who had been enrolled in a randomized controlled trial of VDOT and usual care (directly observed therapy) from July 2019 to December 2021 [17]. A collaborative effort involving researchers at the Makerere University School of Public Health, the University of Georgia, and the Uganda National TB Program staff was key to the success of this study. Kampala city, the capital of Uganda, has an estimated population of 2 million people and has the highest incidence of TB [5]. The diagnosis, treatment, and care for TB are government-funded and provided free of charge to all patients in Uganda. Specifically, in Kampala, TB care is delivered by public health facilities and designated private clinics, which are all regulated by the Kampala Capital City Authority.

### Study Design and Sampling

A cross-sectional exit qualitative study was conducted on a subset of participants exiting the VDOT study in April to July 2021. A subsample of 30 purposively selected participants was included from the VDOT users in the parent study. We ensured wide representation by including equal numbers of men and women; age categories; level of adherence categorized as low, moderate, and high; and HIV infection. We created adherence categories based on the calculated overall medication adherence at the exit of the study: low was <70%, moderate was 70%-<85%, and high was 85%-100%. For example, if 2 participants were of the same sex and had the same level of adherence but if one of them was HIV-infected, we selected that person to represent views of a TB-HIV coinfected individual (see Multimedia Appendix 1 for a summary of the study recruitment plan). The final number of participants to be interviewed was determined based on the attainment of thematic saturation, where additional interviews did not yield new information on the topics of interest [23].

### Detailed Description of the Enhanced VDOT System

**Figure 2:**
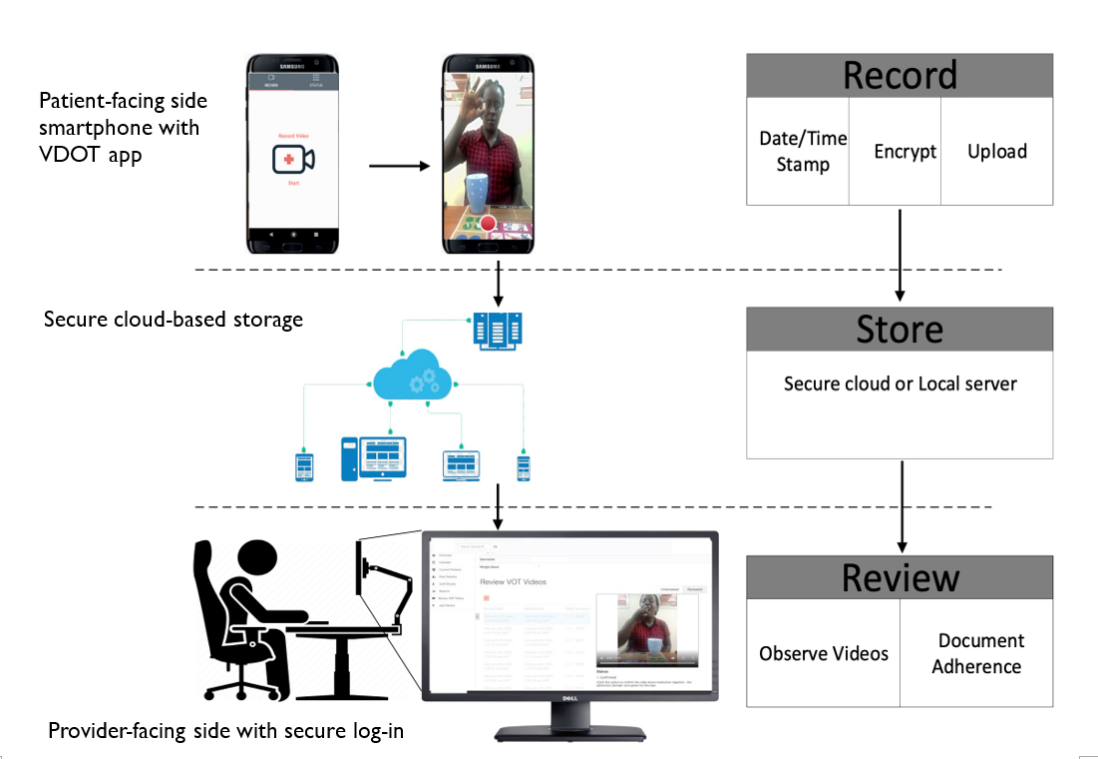
Schematic diagram of the video directly observed therapy system for monitoring tuberculosis treatment. VDOT: video directly observed therapy.

VDOT is a system that is used for asynchronous monitoring of treatment adherence. It has 3 main elements: a patient-facing side that involves a smartphone app for recording and submitting videos; a provider-facing side (the term provider in this study refers to any health worker who uses the VDOT system, either nurse or clinician) that provides a computer-based log-in system from which submitted videos are watched to confirm adherence; and a secure Health Insurance Portability and Accountability Act–compliant cloud server that stores the encrypted videos for review (Figure 2). This system allows for the monitoring of daily doses and the creation of individual and aggregated reports. Additionally, the system generates automatic message reminders that are sent to patients’ phone numbers at the time of the first dose. We adapted the original VDOT system [16] by translating the generic text messages reminders to Luganda language (local dialect). The messages stated that “It’s time to take your medication and record your video.” We added a second text message reminder, which was prompted if a video was not received by the system within 8 hours of the first message reminder. We also enhanced the original VDOT by adding weekly internet data subscription incentives of airtime as supporting elements.

### Participant Recruitment, Data Collection, and Exit Interview Procedure

Eligible participants were patients aged 18-65 years who had participated in the VDOT randomized controlled trial and completed 6 months of treatment. Written consent was required and obtained from all participants separately for their participation in the exit interview. At the beginning, the moderator reviewed the purpose of the interview and reminded participants of their option to withdraw at any time. Structured interview guides were pretested and revised, and a final version was selected for data collection purposes. The questions asked were structured around the constructs of interest, that is, experienced usefulness, ease of use, barriers, and facilitators, and any other challenges encountered as part of the experiences while using VDOT. Interviews were conducted in-person and audio-recorded by a trained team qualitative researcher in the participant’s preferred language (see Multimedia Appendix 2 for interview guides).

### Qualitative Data Processing and Analysis

Audio recordings were transcribed verbatim by the social scientist team that collected the data (VK, PK, and Halima Nakato) within 48 hours to ensure data integrity. Transcripts were reviewed a second time while listening to audio recordings to ensure accuracy. Transcripts were written up by the research team and then reviewed by the 2 study principal investigators. If discrepancies were identified, the transcripts were updated with corrections. The lead social scientist (VK) and 2 trained graduate students (AMD and Gbemisola Tosin) conducted the data coding from the transcripts. We used a hybrid process of deductive and inductive coding to identify content related to the experience of study participation with VDOT. Codes were organized into themes and subthemes, with relationships between themes used to develop overarching categories based on the TAM-RLS constructs. The parent and child codes were organized in the Dedoose 8.1 software by AMD for easy data management, verification, interpretation, and conclusions for the team (see Multimedia Appendix 3 for a sample output from Dedoose). Using Dedoose, we also generated simple statistics to show how frequently a code showed up for each participant. This process was verified by the investigators. The final emergent themes and interpretations were reviewed by our study team members to generate consensus of the main conclusions. The Consolidated Criteria for Reporting Qualitative Studies guidelines were used to inform the reporting of study results [23]. Data from the exit interview and baseline interview were summarized as frequencies, means, percentages, and standard deviations. Quotes were selected based on published recommendations [24]. For example, the quotes selected had to be illustrative of the major experiences conveyed by the respondents. Quotes had to be succinct, yet long enough to convey the point being addressed to the reader. Finally, the quotes selected were representative of strong patterns in data, and quotes of minorities were only included if they provided pertinent information that was important to the theme being assessed [24].

## Results

### Characteristics of the Participants

A total of 30 interviews were conducted among VDOT users. The average age of the VDOT users was 35.2 (SD 12) years. There was an equal distribution of males and females in the exit interview. Approximately 73% (22/30) of the users owned phones, with 45% (10/22) owning smartphones. All cellphone owners made phone calls but differed in other uses such as sending or receiving mobile money, text messaging, and social media. The average duration of the interviews was about 43 minutes with a range of 26 minutes to 66 minutes. The detailed information of the baseline characteristics of the participants is provided in Table 1.

**Table 1:** Baseline characteristics and technology experience of exit interview participants in Kampala, Uganda, in 2021 (N=30).

Demographic characteristics		Values
Age (years; range 18-60), mean (SD)		35.2 (12)
**Sex, n (%)**		
	Male	15 (50)
	Female	15 (50)
**Highest education level, n (%)**		
	None/primary (1-7 years of school)	13 (43)
	Senior (9-13 years of school)	11 (37)
	Diploma/tertiary	6 (20)
**Marital status, n (%)**		
	Never married	13 (44)
	Currently married	10 (33)
	Previously married	7 (23)
**Cellphone ownership, n (%)**		
	Yes	22 (73)
	No	8 (27)
**Type of cellphone (n=22), n (%)**		
	Smartphone	10 (45)
	Other	12 (55)
**Cellphone experience (n=22^a^), n (%)**		
	Make phone calls	22 (100)
	Send text messages	14 (62)
	Send/receive mobile money	21 (91)
	Social media/WhatsApp	17 (77)
**HIV status, n (%)**		
	Positive	9 (28)
	Negative	21 (72)

^a^Overall, total is greater than 22 due to multiple responses.

### Overview of the Results From VDOT Users

For most participants, VDOT was acceptable and easy to use, and they were satisfied with the method of treatment monitoring upon exit from this study. Some VDOT users reported the challenges that they experienced while using VDOT because of lack of technology literacy and privacy and confidentiality concerns. However, after receiving training from the health workers on how to use the system, all participants reported being very satisfied with VDOT. They noted that VDOT helped them to adhere to their medications. The main themes that emerged from the analysis were facilitators and barriers to the acceptability and use of VDOT. These themes are organized and presented under constructs adapted from the TAM-RLS model, including experienced usefulness, experienced ease of use, external facilitators and barriers, and social influence.

### Facilitators of Experienced Usefulness of VDOT

There were several facilitators of acceptance and use of VDOT. Several subthemes were derived under the experienced usefulness construct. These included effective automatic SMS text message reminders, easy reporting of medication adherence, and enhanced patient-provider communication. Specifically, the individuals who were HIV-infected also reported an additional benefit of enhancing their adherence to antiretroviral medications.

#### Effective Text Message and Self-Initiated Alarm Reminders

All participants reported that the text message reminders were instrumental in motivating and maintaining daily adherence. Additional reminders were sent if medication videos were not received within a specific timeframe.

…I liked them (text messages) very much especially because they came at 6 AM. When I got pregnant, I became a bit weak, but whenever I heard that message, I would just wake up because I needed to take my medicines. For me, the message acted as an alarm to be able to wake me up and take my medicines.

Participant #128, female

In addition, several participants also leveraged the alarm feature on the smartphone although it was not a part of the initial VDOT intervention. Alarms were added to further enhance the effectiveness of the reminder system, thus facilitating a positive experience with using VDOT.

…also, the alarm that was set up helped me a lot. Whenever I heard it, even if I did not want to, I would wake up and go and take my medicines. Everyone around the house would also remind me that the alarm had sounded and that I should go and take my medicines.

Participant #129, female

#### Useful for Confirming Medication Adherence

Patients also viewed VDOT as an easier and more convenient way of taking their medication since they no longer had to travel to health facilities regularly compared to when they were under the usual care (directly observed therapy) program. In routine TB program care, patients often take medicines on their own (self-administered treatment) and then self-report adherence at monthly clinic visits. In such situations, patients found the VDOT system extremely useful for confirming via the video evidence to the health workers that they were swallowing their medication.

…I liked the video very much because it helped the doctors to get the proof that they wanted by giving me the phone. The phone helped me very much for them (health providers) to know that I took the medicines that they had given me.

Participant #104, male

#### Timely Patient-Provider Communication and Support

All VDOT users who were interviewed expressed being very happy with the ability to communicate with their health care providers during the video-recording sessions. When patients reported concerns such as the side effects they experienced or asked questions during the video sessions, they received a follow-up phone call on the same day from the health workers. Patients highly valued receiving the necessary counseling, education, or reassurance from the health workers during the follow-up phone calls. Moreover, the patients pointed out that VDOT was very useful because they felt that the health providers were able to monitor their daily progress and physical well-being almost in real-time as they watched their videos.

…also, the care that the doctors (health workers) gave me was always good. For example, whenever I had any side effects, I would report them in the video and the doctors (health workers) called me back immediately. I would explain to them the problem, and they would tell me not to worry as things will get better; so, the care they gave me was very good.

Participant #31, male

#### Supports Antiretroviral Therapy for TB/HIV-Coinfected Patients Using VDOT

Some participants who were TB/HIV-coinfected reported that VDOT provided an additional benefit of enhancing adherence to their antiretroviral treatment. This usefulness was noted because the VDOT intervention was originally set up for monitoring TB medications.

…and the good thing is whenever I was taking the TB medicines I could also take the HIV medicines at the same time, which helped me and my life very well.

Participant #78, female

### Facilitators of Experienced Ease of Use

The perceived ease of use was mostly influenced by technology literacy and smartphone skills. For example, all participants had prior cellphone use experience, but only 45% (10/22) had experience using a smartphone. However, from the exit interviews, it seemed that an initial lack of smartphones had a very minimal effect on the overall ease of use of VDOT. Regardless of prior smartphone use, most participants reported that the VDOT app was user-friendly.

#### Technology Usability Training and Support

One key factor that improved the ease of use of VDOT was the training support that patients received before and during this study from the health workers. At the initiation of this study, participants randomized to the VDOT intervention had a thorough training session to guide them through the step-by-step process of using the VDOT app, recording, and submitting their medication videos. This was followed by a practice session between the patient and health provider. The participants were offered unlimited access to retraining if they encountered difficulties with any aspect of the technology until they were proficient. Most participants mastered VDOT use after the training sessions and were able to record and send videos.

…The fact that the doctor (health provider) taught me how to use it (VDOT) helped me a lot, and when she taught me, I also took time to understand how to use it on my own. This way, I followed her exactly and found myself learning how to use it.

Participant #128, female

#### Family and Community Social Support

The continuous access to social support from family members, friends, and neighbors during the treatment and video-recording process positively impacted the ease of using VDOT. Some participants reported that family members were very supportive of their use of the VDOT system and adhering to their medications. Some older patients who lacked technology literacy reported that they received support to record their videos from family members who had experience with smartphones. Moreover, some participants cited that the SMS text message reminder notifications and alarms would prompt family members and even neighbors to urge the patients to record their videos.

…whenever I heard the alarm (reminder), I would wake up, prepare everything that I’m going to use, and then my son or neighbor would video record me while I’m taking my medicines.

Participant #31, male

…They (family members) could tell me “do not worry, TB is curable; just take your medicines on time and the rest will be okay.” They also told me to be patient and not to fear. This gave me more courage to take my medicines on time since I had some people (family) backing me up and encouraging me.

Participant #97, female

#### Incentives to Motivate Adherence

The provision of weekly incentives in the form of airtime minutes and internet data facilitated the easy use of VDOT. Patients were highly motivated to continue adhering to their prescribed medications as well as sending videos to the VDOT system. Although the weekly incentive was a small amount (~US $0.33), participants repeatedly expressed that it had positively facilitated their VDOT experience and enhanced access to family social support. The weekly incentives enabled patients to make phone calls to contact their caregivers in case of any needed support rather than keeping them tied down at home.

…Airtime kept me connected to my family members. I was not earning because I was sick and I could not work. So I used the airtime to call my friends to send me some money to buy food because it is hard to take drugs when you are hungry.

Participant #90, male

…I used the phone to communicate with my husband. He is a boda boda rider; he used to ride back home to check on me. When I got a (study) phone, he would check on me (by calling me on the phone) while he was at his work; so, it also helped him to be at work instead of having to ride back home.

Participant #50, female

### External Barriers to the Usefulness and Ease of Use for VDOT

Although all participants reported that VDOT was acceptable, they highlighted some important challenges that they experienced during its use. These barriers are based on constructs from the TAM-RLS, as described earlier. The major themes that emerged were related to inadequate supportive technical infrastructure. The subthemes considered under technical infrastructure were unstable electricity, poor cellular connectivity, and technology errors or failures.

#### Unstable Electricity

Some participants reported unstable flow of electricity in their homes that affected the charging of the smartphones and the access to lighting needed for recording the medication videos early in the morning. Those patients had to rely on friends or neighbors to charge the study smartphones. Some patients reported navigating the lack of light by recording their videos outdoors, which was quite inconvenient. Therefore, unstable electricity negatively affected both the ease of use and the usefulness of the VDOT system.

…sometimes I did not record myself and when the doctors (health providers) called me and asked me about the video, I told them I was not able to record myself because we did not have power nor did we have network. However, I did take the medicines, and that is what I told them.

Participant #68, male

#### Cellular Network Connectivity

Some participants occasionally experienced issues with cellular network connectivity, which delayed them from sending videos, thus disrupting the usefulness and purpose of the intervention. However, it is important to note that the VDOT system had a built-in feature that overcame this challenge. In the absence of cellular network connectivity, videos could still be recorded and stored in the phone. When better cellular network was available, the videos were then automatically sent to the cloud server.

…Another issue was that of network connectivity. I would send a recording, but it was never received. Then the doctors would start calling because they haven’t received the recording.

Participant #69, female

#### Technological Failures

A small number of participants reported encountering problems with smartphone app errors or malfunctioning. This resulted in either a video not being recorded or patients having to delay taking their medication. The technological failures that occurred became a barrier to both the usefulness and ease of use of VDOT. Another minor but critical failure that occurred less frequently was the automatic text message reminders, which sometimes did not turn off after a video was successfully submitted. This resulted in patients erroneously receiving unnecessary reminder messages to submit videos, which were deemed inconvenient by the patients. These technological failures or errors were relatively easy to fix once reported by the patients.

…They should stop sending messages after we have taken our medicine. You can take your medicine in the morning, and you know it is private. Then, messages will come again when you are out with friends. They (friends) can start to wonder what medicine you are taking that needs messages.

Participant #56, male

#### Stigma, Disclosure, Privacy, and Confidentiality

Patients expressed some perceived and experienced negative effects when using VDOT. For example, a small number of participants perceived stigma or a threat to breach of privacy and confidentiality of the videos that they submitted to the VDOT system. The main fear expressed was that of unintended disclosure of their disease status to family or non–family members. Other participants reported skipping their medication because they lacked a private place and were uncomfortable recording videos in the presence of their family members because they had not disclosed their disease status. Only a few participants experienced stigma when family members ridiculed them for taking TB medications and using VDOT.

…and yet I did not want to always take medicines for outside people to know that I was sick. I lost my father (had to travel for the funeral), and it was difficult for me to get my medicines and to video record when I was in public. Therefore, for those 2 days, I did not video record myself.

Participant #129, female

Some respondents also reported experiencing negative social influence from family members who discouraged or ridiculed them for taking their medicine saying that their illness would be exacerbated. Other family members falsely insinuated that the medication videos would be misused and shared on public media channels. However, the health workers combated this misinformation through education and reassurance that principles of confidentiality were upheld.

…When it came to my siblings, they thought it (VDOT) was a scam and that the videos would be made public. Therefore, they did not welcome the idea in the beginning. Even toward the end, they still believed it was a scam, but then they became more accepting since I recorded the videos in front of them.

Participant #69, female

## Discussion

### Principal Findings

This study used qualitative exit interviews to evaluate the experiences of female and male patients with TB using VDOT during their treatment in Kampala, Uganda. To our knowledge, this is the first study that reports on user experiences with VDOT in Africa. We adapted the TAM-RLS conceptual model to guide the analysis of the experienced usefulness and ease of use for VDOT. We found that most participants reported VDOT as acceptable, useful, and easy to use. The facilitators of positive experience with VDOT were the effective text message reminders, convenience of adherence confirmation, enhanced provider-patient communications, technology training support by health care providers, and family support. Patients with TB/HIV coinfection experienced a dual benefit of adherence support for TB medications and their antiretroviral therapy. The main barriers to VDOT’s usefulness and ease of use were related to inadequate technical infrastructure and negative social influences such as fear of unintended disclosure of disease status and stigma by family members. Fears of breaches in privacy and confidentiality arising from the misuse of the videos also emerged as potential barriers to the use of VDOT. We identified prominent subthemes that provide insights into facilitators and barriers that could inform future adoption and scale-up of VDOT in the local Ugandan context. The findings of this study are generally consistent with results from our previous study that explored stakeholders’ perceptions about VDOT in Uganda [25].

The experienced usefulness of VDOT was facilitated by several aspects. For example, patients reported that the daily automatic text message reminders were effective in supporting their adherence to TB medication. This finding is consistent with previous studies that have reported higher adherence using digital monitors with reminders compared to the usual directly observed therapy care [26,27]. Patients using digital monitoring systems that utilize message reminders have shown high acceptability, particularly because they facilitate formation of a routine in taking their medications, especially amid busy schedules [28]. Interestingly, some patients also utilized phone alarms to enhance the text reminder system that was already in place as part of the intervention, suggesting that they were eager to take their medications. A few patients expressed concerns that the text message reminders could pose a threat to privacy, leading to unintended disclosure of one’s disease status if they were accessed by another person. Previous studies conducted in similar resource-limited settings have also reported that patients perceived being stigmatized even though they had found the text reminders helpful [28-30]. In order to address concerns of accidental disclosure of disease status in our study and other studies, the text reminders messages did not specify sensitive information [28]. The frequency and timing of text messages can raise concerns with some patients. For example, repeated text reminders have been perceived as being overly intrusive in some settings [30,31], and therefore, patients’ preferences should be considered.

The usefulness of VDOT was reportedly enhanced when patients were able to conveniently confirm medication adherence and have timely interactions with their health care providers. The patients also expressed a feeling of being closely cared for because of the moral support, health education, and side effects monitoring that they received albeit remotely. This finding from users’ experiences was in contrast to that of our previous study of stakeholders in Kampala, where users perceived that the lack of in-person interaction in VDOT would negatively affect the patient-provider relationship [25]. This improved relationship was further facilitated through follow-up phone calls initiated by the health workers to offer appropriate support to patients who had reported concerns in the video or had missed submitting videos. VDOT also facilitated timely patient support that was prompted by daily visualization of the patients’ general condition through the recorded videos. In another previous study, VDOT was perceived to enhance health care provider–patient communication and impact patient engagement with their treatment [8]. The consistency in these positive aspects makes VDOT a promising patient-centered approach for TB disease management. Moreover, similar findings were reported in HIV studies conducted in rural Uganda that showed that participants viewed increased or real-time support from health workers as being cared for and can be a factor that increases adherence to HIV medication [32]. In addition, Campbell and colleagues [33] found that both instrumental and emotional support often arose through the close relationships that participants formed with research staff and with each other. This suggests that study-derived social support motivated some participants to adhere to antiretroviral therapy [33].

The experienced ease of use was related to technology training support in the use of smartphones and the VDOT app. The lack of technology literacy was previously cited in our study as a perceived barrier to using of VDOT, especially for older adult patients in Uganda [25]. In our study, all patients received thorough technology training at enrollment and continuous support through the training received from health workers. As such, participants found VDOT easy to use, regardless of education level or age. Our findings are consistent with more recent studies conducted in both urban and rural settings that suggest that with training, age does not significantly affect the ease of use of VDOT [12]. Therefore, we recommend that training and continuous technology support should be an integral part of adoption and scale-up for future VDOT programs.

Family social support was also key to the ease of use experienced with VDOT by the patients in our study. Most patients reported receiving strong moral support from their family members when they needed to take medications and record videos. A few older patients who were not familiar with smartphones and apps received some support from their family members. This helped to enhance the technology training they had received from the health providers. This finding suggests that family members can be an integral part of the treatment process and should therefore be involved to the highest extent possible when using VDOT and other digital technologies to motivate treatment adherence. Lack of social support is a well-known determinant of poor adherence to treatment of TB [34] and HIV [29], especially in low-resource settings.

Monetary and nonmonetary incentives were provided in the form of mobile-delivered money, phone airtime calling minutes, and internet data bundles in our study. Participants highlighted that the incentives were important facilitators of ease of use for VDOT. This finding is consistent with previous studies that have shown that cash incentives may generate a desired behavioral response for a relatively small price [35]. Moreover, low-income populations may be more responsive to cash incentives compared to other populations. In our study, we offered US $0.33 as a weekly cash incentive for 7 consecutive video submissions, and VDOT users showed higher treatment adherence compared to patients under usual care. A study in Brazil concluded that a conditional cash transfer program can contribute to improvement in TB cure rate [36]. A study in Moldova found that provision of incentives to patients with TB significantly improved treatment success rates and retention [37]. Similarly, a recent study in Uganda found that patients with TB who received an incentive of US $1 were more likely to be cured and less likely to be lost to follow-up compared to those who did not receive the incentive [38]. We did not find any published studies on the relationship between incentives among patients with TB using digital adherence technologies and treatment outcomes; therefore, further research is needed on this topic.

External barriers to the experienced usefulness and ease of use were largely related to inadequate technical infrastructure to support the VDOT system. Unstable electricity, poor network connectivity, or technological failures affected some patients’ ability to use VDOT smoothly, thereby leading to missed videos. This finding is not surprising given that this study was conducted in a low-resource setting and is consistent with that reported in our previous pilot study [14]. Previous studies conducted in similar settings have reported failure to charge phone batteries due to lack of electricity as one of the commonest issues faced by patients when using digital monitoring systems [28,29]. Low-cost solar power banks for charging smartphones could be a short-term solution to alleviate the challenges of unstable electricity. For poor cellular network connectivity, the VDOT system has a fail-safe feature, where recorded videos are stored and submitted automatically when the network connection is restored. This feature ensures that medication doses could be verified even in the absence of constant network connection. The users need reassurance and awareness about this built-in functional feature that saves the videos.

Perceived or experienced concerns about stigma, disclosure privacy, and confidentiality were expressed in our study. Specifically, a few patients expressed perceived breaches in privacy and confidentiality citing major fears of misusing the videos in the public media by health workers or family members, leading to unintended disclosure of disease status. To our knowledge, such a situation did not arise in our study. Stigma and other negative influences were experienced by a few patients who reported being ridiculed by either family or community members for accepting to record videos of themselves while taking TB medications. Similar concerns were reported in our previous VDOT qualitative study [25] and with other studies across various settings [26,39,40]. It is important to note that the specific effect of VDOT on stigma, privacy, and confidentiality remains unclear because previous VDOT studies have reported mixed findings [16,39]. The lack of a definitive direction of effect of VDOT could be explained by the diverse cultural understanding of these concerns in different settings. To minimize feelings of mistrust, patients should be continuously reassured about the secure storage of the videos. Providers should also strive to understand and address the cultural perceptions of privacy and stigma.

### Strengths and Limitations

This study had some strengths, for example, we used TAM-RLS, which was developed and first validated in a Ugandan population of persons living with HIV/AIDS and on antiretroviral treatment. This model enabled us to assess the experienced facilitators and barriers of VDOT’s usefulness and ease of use. This study is based on participants from a randomized trial, which minimized the volunteer bias that could lead to overestimation of VDOT acceptance. Furthermore, to increase the representation of experiences, we purposively stratified the sample of participants in the exit study to ensure equal numbers by sex, variation in adherence levels, and HIV status.

Our study findings have some limitations. First, the reported positive experiences are not generalizable to all patients with TB because our study provided a free smartphone, internet data, and airtime incentives, which themselves can serve as motivators to adherence and acceptance. In resource-limited settings like Uganda, TB programs are not likely to provide all the technology and supportive services that our study participants received; hence, the experiences of routine patients could differ. In this study, we did not compare the experiences of VDOT users with usual standard of care, which could help to identify subgroups of patients with TB who might prefer one method over the other.

### Implications for Adoption and Scale-Up of VDOT in Low-Resource Settings

The promise of VDOT as one of the digital adherence technologies is reinforced by positive experiences reported by users in a low-income setting. Successful adoption and scale-up of VDOT will require investment and identification of resources at the health care provider, health system, and patient levels. At the health care provider and health system level, there is a need for upfront investment in the local technology infrastructure, including computers, stable electricity, and skills training to build proficiency in the use of the VDOT system. Private-public partnerships between telecommunication companies’ and the Ministry of Health’s National TB program could support resources for VDOT scale-up. For example, telecommunication companies could use some of their corporate social responsibility to support internet subscription for a specific number of patients each year. In addition, a broader plan to integrate the VDOT system into the Health Management Information System could strengthen monitoring for multiple diseases, including HIV/AIDS treatment adherence. At the patient level, community resources such as personal smartphones could be leveraged with a goal to encourage utilization in delivering the needed support. Our study showed that 45% (10/22) of the patients owned smartphones. Given the current trends of technology adoption, smartphone ownership and experience is likely to grow, thus facilitating more use of app-based interventions like VDOT in populations. Indeed, the COVID-19 pandemic highlighted the necessity of remote delivery of health care and patient support. A new trend in telehealth has followed, making it self-evident that efforts toward building a strong technology-based monitoring system will yield future benefits for patients and health care providers in the long term.

### Conclusions

Overall, participants had positive experiences with the enhanced VDOT. They found the enhanced VDOT system user-friendly, beneficial, and acceptable, particularly due to the supportive features such as SMS text message reminders, incentives, technology training by health care providers, and family support. However, it is crucial to address the perceived privacy, confidentiality, and stigma concerns related to VDOT. Upstream investment in the local infrastructure is necessary to address technology-related barriers that could limit the implementation and scalability of VDOT.

## Appendix

Multimedia Appendix 1 Video directly observed therapy exit interview recruitment guide.

Multimedia Appendix 2 Translated exit interview guide.

Multimedia Appendix 3 Sample output from Dedoose software.

## Abbreviations

TAM-RLS: Technology Acceptance Model in Resource-Limited Settings
TB: tuberculosis
VDOT: video directly observed therapy
WHO: World Health Organization
