# Supplementary material for: Acceptability, Usefulness, and Ease of Use of an Enhanced Video Directly Observed Treatment System for Supporting Patients With Tuberculosis in Kampala, Uganda: Explanatory Qualitative Study
Source: JMIR Form Res. 2023 Nov 10;7:e46203. doi: 10.2196/46203 (PMC10674141; doi:10.2196/46203)
Supplement: Multimedia Appendix 1 [file formative_v7i1e46203_app1.pdf]

VDOT Exit Interview Study Recruitment Guide

Characteristics of Exit Interview participants

**Male**	Age	Age	Age	Subgroup total	**Female**	Age	Age	Age	Subgroup total	
**Age & adherence performance**	18-30 y	31-50	51+	Total	**Age & adherence performance**	18-30 yrs	31-50	51+		**Total**
Low: <70%	2	2	2	6	Low < 70%	2	2	2	6	
Moderate: 70-<85%	2	2	2	6	Moderate (70 to <85%)	2	2	2	6	
High: 85%-100%	2	2	2	6	High (85% to 100%)	2	2	2	6	
Other characteristics				18	Other characteristics				18	36
