# Supplementary material for: Acceptability, Usefulness, and Ease of Use of an Enhanced Video Directly Observed Treatment System for Supporting Patients With Tuberculosis in Kampala, Uganda: Explanatory Qualitative Study
Source: JMIR Form Res. 2023 Nov 10;7:e46203. doi: 10.2196/46203 (PMC10674141; doi:10.2196/46203)
Supplement: Multimedia Appendix 2 [file formative_v7i1e46203_app2.pdf]

Makerere University/University of Georgia (UGA)

DOT Selfie Study Patient's exit interview (VDOT ARM)

PATIENT IN DEPTH INTERVIEW GUIDE (Luganda).

Introduction.

Nkulamusiza Nyabo/Ssebo, erinya nze ....nkola nabasawo mukunonyereza okwa DOT selfie wansi wa Makerere University School of Public Health wamu ne University of Georgia okuva mu America. Nkwebaza okuwayo obudde ne wewaayo kyeyagalire okwetaba mukuddamu ebibuuzo mungeri ey'emboozi. **Ebitonotono ebikwata ku byetugenda okukola olwaleero:** Amaloboozi gaffe mukunyumya kwaffe kujja kukwatibwa kukatambi oluvanyuma biwandiikibwe naye erinya lyo terijja kulabikira kukiwandiiko kyonna ekikwata kumboozi eno. By'oyiseemu, by'otesa nebirowozobyo ku nkola yokukwata akatambi anga omira eddagala byamugaso nyo era tukusaba obeere waddembe okuwa endowoozayo. Era kyamugaso okujjukira nti buli kimu ekigenda okwogerwa wano kyakyaama era nti tekijja kugabanyizibwaako namuntu yenna ali wabweru wokunonyereza kuno. Olina ekibuuzo kyonna nga tetunaatandiika?

Background.

Tutandiike nawe nga ombulira ebitonotoono ebikukwatako.

Emyaka gyo?
Obeera nani?
Okola ki okufuna sente?

Enkola yokukwata akatambi nga omira eddagala yakuwebwa etya?

Wawulira otya nga owereddwa enkola yokwekwata kukatambi nga omira eddagala?

Abakozi mukunonyereza bakugamba ki?

Wawulira nga olina eddembe okukiriza oba okugana enkola eyokwekwata kukambi?

Wali wawulirako enkola yokwekwata kukatambi nga omira eddagala?

Oba ye, wagiwulira wa?
Kiki kyewalowoozaako bwewasooka okuwulira kunkola eno?

Technology literacy

Okusinzira kuby'oyiseemu nga okozesa amasimu, kiki ekyakifuula ekyangu eri gwe okwekwata kukatambi nga omira eddagala lyo erya kafuba? **Kiki ekyasinga okukuyamba?**

Makerere University School of Public Health
Valid Thru:
25 FEB 2022
Higher Degrees, Research and Ethics Committee

Kukusomozebwaki kwewafuna mumukwekwata kukatambi nga omira eddagala? **Kiki ekyasinga okukuwa obuzibu?**

VDOT App

Biki app byekozesa ebyakifuula ekyangu eri gwe okwekwata kukatambi nga omira eddagala lyo erya akafuba?

Wafuna buyambi ki okusobola okukozesa App eyo obulungi?

Kiki kyewasinga okwagala ku VDOT App?

Kiki kyewasinga obutayagala ku VDOT App?

Kusomozebwaaki kwe wafuna mukukzesa VDOT App? **Probe about; time, app frezes and updates if not mentioned.**

Waliwo kyoteesa okulaba nga app ekola bulungi okusingawo?

Reminder SMS

Nga ekimu kubikolebwa mukunonyereza, wafunanga obubaka ku ssimu obukujjukiza buli lunaku.

Kiki kye wasinga okwagala kububaka obwo?

Kiki kyewasinga obutayagala kububaka obukujjukiza?

Owulira otya kukyokufuna obubaka obukujjukiza buli lunaku?

Data bundles and incentives.

Owulira otya ku busaganda bwa data bwewafunanga buli sabiiti?

Kiki kyewasinga okwagala ku busaganda bwa data?

Kiki kyewasinga obutayagala kubusaganda bwa data?

Kyandibadde kyanjawulo kitya singa wali tofuna data oyo gwebakusindikiranga buli wiki?

Kiki ekyabaangawo buli lwewafunanga airtime okuva mubanonyereza? **Probe about motivation to send videos.**

VDOT as a strategy to improve adherence.

Biki byewasubira nga tonatandiika kukozesa nkola yokwekwata kukatambi nga omira eddagala?

Bwewasubira byebyo byewayitamu nga okozesa enkola eno?

Makerere University School of Public Health • Higher Degrees, Research and Ethics Committee • Valid Thru: 25 FEB 2022

Kibaddenga kitya eri gwe okwekwata kukatambi nga omira eddagala lyo erya TB?

Birowoozo ki ebibaddenga bikujjira mukaseera ako nga wekwata kukatambi?

Ani yateranga okubeerawo nga omira eddagala nokwekwata kukatambi?

Okumira eddagala wamu nokwekwata kukatambi wabikolanga mubudde ki? **Why this?**

Oba waliwo eyakulaba nga omira eddagala nga bwekwata kukatambi, wawulira otya nga akulabye?

Oba nga waliwo obuyambi bwewafuna okuva mubantu bobeera nabo, mikwanojjo, abaakulabiriranga, aboluganda lwo oba abasawo okusobola okumira eddagala nokwekwata kukatambi, nyinyonyola kubuyambi obwo. **What are your thoughts about this support?**

Oba nga kyaaliwo, abantu bobera nabo bakuremesanga batya okumira eddagala lyo wamu nokwekwata kukatambi? **Probe about phone sharing, switching sim cards etc.**

Kiki kyewasinga okwagala kunkola yokwekwata kukatambi nga omira eddagala lya akafuba? **Probe for explanation.**

Ekyokwekwata kukatambi nga omira eddagala kyakuyamba kitya okumira eddagalalyo obulungi? **Probe for the benefits of VDOT towards adherence.**

Cell phone offer

Nkimanyi nti akalulu bwekakusuula kunkola eyokukozesa essimu okwekwata kukatambi nga omira eddagala, wawebwa essimu.

Wawulira otya kwekyo?

Abasawo mukunonyereza bakugamba ki kubikwatagana ne ssimu eyo? Wawulira otya kubyebakugamba?

Ba/ani gwe wayogera naye okumugamba nti owereddwa omukisa okutwala essimu? Ba/yakuddamu ki? Wamaliriza okoze ki?

Oba olina omwagalwa, omwagalwaawo yawulira atya kukyokuba nti wafuna essimu okuva ku ddwaliro e Lubaga?

VDOT Challenges and concerns

Okutwalizaawamu, kusomozebwaki kwewafuna mukwekwata kukatambi nga omira eddagala lya akafuba? **Probe about stigma (not want to be seen), time, data and network issues, phone safety among others.**

Makerere University School of Public Health • Higher Degrees, Research and Ethics Committee •
Valid Thru:
25 FEB 2022

Okusomozebwa okwo wakuvunuuka otya?

Walinamu okweralikirira mukuwereza akatambi eri abasawo? **Kweralikirira ki?**

Waali ofaayo kukikula ky'omusawo alaba akatambi ko okugeza mukazi oba musajja? Lwaki?

Mbulirako kulunaku lwotamira ddagala lyo elyakafuba oba kulunaku lwewamira naye notekwata kukatambi. Kiki ekyakiretera okubaawo?

Wawulila otya nga tewekute kukatambi?

During internet closure.

Kyaali kitya eri gwe okumira eddagala wamu nokukwata video mukiseera yintaneti/enkolo yemitimbgano weyagyibwako?

Kiki ekyaali ekyenjawulo kubyabulijjo?

Nkola ki zewwatekawo okulaba nti osigala nga omira eddagala era nokwekwata kukatambi nokawereza abasawo?

Effects of COVID-19

Mbulirako nga bwekiri oba bwekibadde okumira eddagala lya TB mukiseera kino nga COVID-19 wali.

Omugalo gwa COVID-19 gwakyuusa gureseewo ki mungeri gyomiranu eddagala ly'a kafuba. Oba waliwo.

Embeera eno yaretawo ki mukukwata nokuwereza akatabi eri abasawo?

Future recommendations.

Kiki kyewandiyagadde kirongosebweemu oba kikyusibwe mumaaso singa abalwadde basabibwa okwekwata kukatambi nga bamira eddagala?

Experience of study exit.

Wawulira otya kukyokulekerawo okwetaba mukunonyereza? **Probe for any concerns.**

Wawulira otya bwebakusaba okuzayo essimu eri abanonyereza?

Waliwo ekintu ekirala kyewandiyagadde okungamba? Oba olyawo kyetutayogeddeko?

Olinayo ekibuuzo?

Webale nyo obudde bwompadde.

Makerere University School of Public Health • Higher Degrees, Research and Ethics Committee • Valid Thru: 25 FEB 2022
