# Supplementary material for: Acceptability, Usefulness, and Ease of Use of an Enhanced Video Directly Observed Treatment System for Supporting Patients With Tuberculosis in Kampala, Uganda: Explanatory Qualitative Study
Source: JMIR Form Res. 2023 Nov 10;7:e46203. doi: 10.2196/46203 (PMC10674141; doi:10.2196/46203)
Supplement: Multimedia Appendix 3 [file formative_v7i1e46203_app3.pdf]

Partial output from Dedoose software showing parent codes and child codes

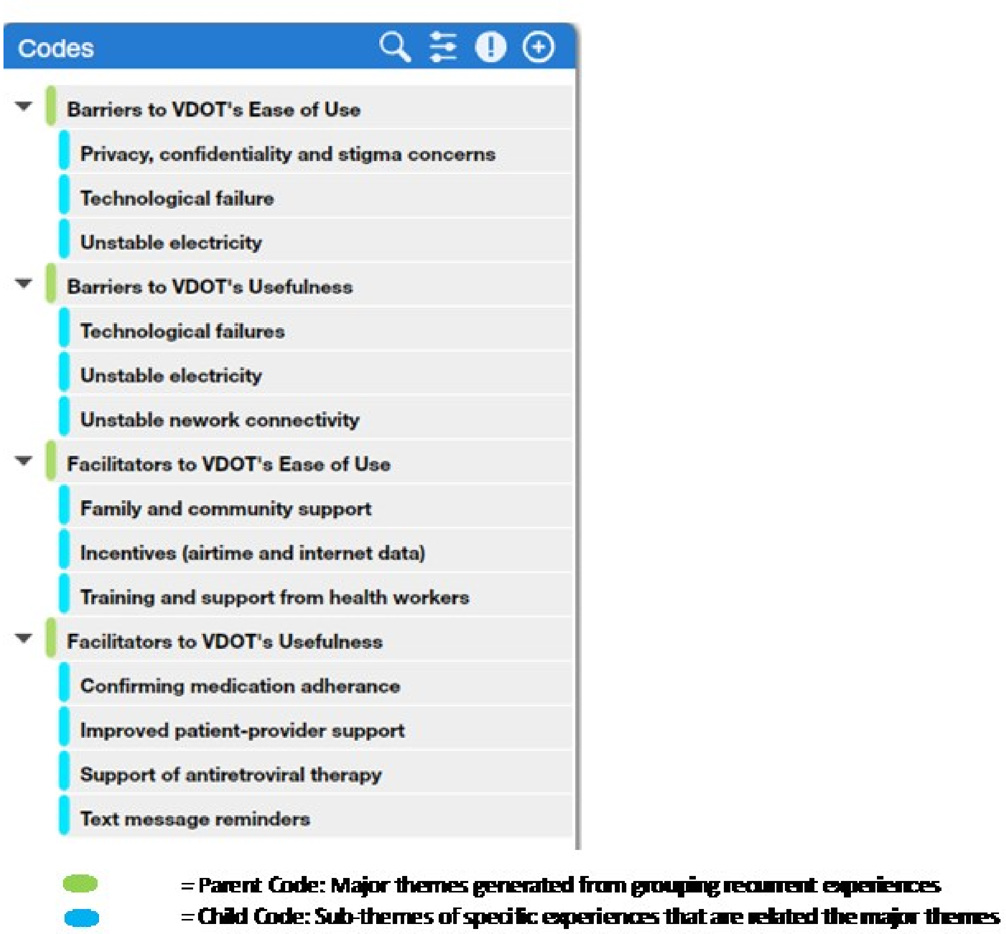

Table showing frequencies for sample parent and child codes from Dedoose software

Parent Code	Child Code	Frequencies
**Ease of use**	Yes, it was easy to use smartphone	25
	No, it was not easy to use smartphone	0
	Training received was effective	18
	Training received was not effective	0
**Barriers to use and acceptability**	VDOT was not initially readily acceptable<br>• Hesitation due to smartphone literacy<br>• Fear of smartphone being lost or stolen<br>• Smartphone literacy	19
